# Neural Basis of Societal Risk for Mental Illness: Focus on Ethnic Minority Position and Racial Prejudice

**DOI:** 10.1192/j.eurpsy.2022.118

**Published:** 2022-09-01

**Authors:** A. Meyer-Lindenberg

**Affiliations:** Zentralinstitut für Seelische Gesundheit, Dept. Of Psychiatry, Mannheim, Germany

**Keywords:** migration, Dopamine, social exclusion, cingulate cortex

## Abstract

Background Urban birth, urban living, and ethnic minority status are established risk factors for schizophrenia, but the mechanisms are unclear. Previous evidence suggests a causal role of social exposures and adverse experiences, but experimental evidence is scarce. Methods We combine multimodal neuroimaging with ecological momentary assessment, geolocation and geospatial analysis in an epidemiological longitudinal sample in Germany. Results We find that established risk factors converge on the perigenual cingulate-amygdala-ventral striatal pathway as shown by structural and functional imaging, supporting a role for the ventral-striatal system in psychosis risk. Using a combination of PET and fMRI data in migrants, we suggest a mechanistic link to psychosis by increased dopamine release and synthesis in striatum secondary to prefrontal dysregulation. Importantly, the regulatory system identified overlaps with that implicated in racial stereotyping and prejudice. Moreover, an experiment measuring information flow during an exchange between migrants and non-migrants indicates that during a trust interaction, cultural distance governs the exchange. Conclusions This work shows a convergent risk circuit related to minority position and migration that could guide primary prevention of schizophrenia through reduction of manifestation risk by contextual intervention.

**Disclosure:**

No significant relationships.

